# Assessment of factors affecting diabetes management in the City Changing Diabetes (CCD) study in Tianjin

**DOI:** 10.1371/journal.pone.0209222

**Published:** 2019-02-12

**Authors:** Jiageng Chen, Xiyue Jing, Xiaoqian Liu, Anna-Mari Volkmann, Yunfeng Chen, Yuanyuan Liu, Dandan Li, Duolan Han, Yuting Guo, Fei Gao, Na Han, Xuying Wang, Haozuo Zhao, Xinjun Shi, Yanan Dong, Liming Chen, David Napier, Jun Ma

**Affiliations:** 1 School of Public Health, Tianjin Medical University, Tianjin, China; 2 University College London, London, United Kingdom; 3 Metabolic Diseases Hospital, Tianjin Medical University, Tianjin, China; Mexican Social Security Institute, MEXICO

## Abstract

**Objective:**

This study aimed to identify the local levels of vulnerability among patients with Type-II diabetes (T2DM) in Tianjin. The study was aimed at curbing the rise of T2DM in cities.

**Methods:**

229 participants living with T2DM were purposively sampled from hospitals in Tianjin. Collected data were coded and analysed following well-established thematic analysis principles.

**Results:**

Twelve themes involving 29 factors were associated with diabetes patients’ vulnerability: 1. Financial constraints (Low Income, Unemployment, No Medical Insurance/Low ratio reimbursement); 2. Severity of disease (Appearance of symptoms, complications, co-morbidities, high BMI, poor disease control); 3. Health literacy (No/Low/Wrong knowledge of health literacy); 4. Health beliefs (Perceived diabetes indifferently, Passively Acquire Health Knowledge, Distrust of primary health services); 5. Medical environment (Needs not met by Medical Services); 6. Life restrictions (Daily Life, Occupational Restriction); 7. Lifestyle change (Adhering to traditional or unhealthy diet, Lack of exercise, Low-quality sleep); 8. Time poverty (Healthcare-seeking behaviours were limited by work, Healthcare-seeking behaviours were limited by family issues); 9. Mental Condition (Negative emotions towards diabetes, Negative emotions towards life); 10. Levels of Support (Lack of community support, Lack of support from Friends and Family, Lack of Social Support); 11. Social integration (Low Degree of Integration, Belief in Suffering Alone); 12. Experience of transitions (Diet, Dwelling Environment).

**Conclusion:**

Based on our findings, specific interventions targeting individual patients, family, community and society are needed to improve diabetes control, as well as patients’ mental health care and general living conditions.

## Introduction

Diabetes mellitus (DM) now affects one in 12 people globally[1]. Over the last decade, China has become the leader in the global DM epidemic[2, 3], and prevention and management of DM have become critical public health issues in China[4]. The increased number of DM patients has also resulted in the appearance of substantial economic losses for the patients and their families, along with those of health systems and national economies through direct medical costs and loss of work and wages[5].

The prevalence rate of diabetes increased from 6.4% in 2000 to 9.5% in 2010, with a 12% increase of rate of urbanization in the same period of time in Tianjin[6]. The healthcare system in Tianjin is trying hard to accommodate multi-centred approaches to manage diabetes patients’ conditions, but the outcomes remain unsatisfaction[7]: Only 50% of the diagnosed patients received subsequent medical care. Among the population receiving care, only 24% achieved the treatment target. These figures were far worse than the general indications in the Rule of Halves framework (The Rule of Halves is a restatement of the meaning of 'median' in statistics—in any population, and using any measure, half the people will be on one side of the median, half on the other.) within which diabetes and some other chronic diseases operate (cancer, heart disease, high blood pressure) [8].

Tianjin joined in the Cities Changing Diabetes (CCD) program, collaborating with 5 other cities to fight against urban diabetes. The aims of CCD are to issue a wake-up call in cities regarding the inevitability of urban diabetes, to push urban diabetes higher on the global health agenda and to inspire local actors across the world. In line with global aims, this manuscript was aim to explore the culture- and context-specific research on local vulnerability for type 2 diabetes (T2DM) and provided suggestive insights for urban diabetes control.

## Methods

### Methodological approach

An open qualitative approach that aims at discovery instead of hypothesis testing helped to explore the range, depth and complexity of the intermediary factors that impact T2MD patients’ vulnerability. Therefore, the study was conducted as a multi-centre, cross-sectional qualitative study in Tianjin. The transcribed interview data were analysed in accordance with the rule of Thematic Analysis (TCA) [9]. TCA is the most common form of analysis in qualitative research, most researchers considered TCA to be a very useful method in capturing the intricacies of meaning within a data set for it focuses on examining themes within data. TCA is a systematic data reduction methodology can extract the most salient information from large numbers of qualitative interviews, then ready for analysis. Systematic data reduction ‘sharpens, sorts, focuses, discards, and organizes’ data in order to drawn and verify the “final” conclusions through in-depth coding of salient data. The coding process is a central feature of qualitative data analysis as it identifies and makes manageable the precise information needed to answer a stated research question. It is also the primary process for developing themes within the raw data by recognizing important moments in the data, then encoding it prior to interpretation [9]. This framework is suitable for capturing the subjective experiences of patients with T2DM within their own social and cultural context and reviewing them with a view towards caring and understanding the perception of patients throughout the process of healthcare.

### Field workers’ enrolment

A total of 26 groups of field workers were involved in the interview. They are experts from various well-known hospitals in Tianjin. (S1 Table) All field workers had been trained before the interview. The semi-structured open-ended questions were undertaken with participants enrolled by the field workers. A final total of 259 participants were included in the research, of which 219 interviews occurred in hospital, 37 in participants’ homes and 3 in cafes. Field workers audio-recorded each interview, wrote up the executive summary of their observations, and collected the demographic and clinical information of each participant.

### Sample size

To capture the most vulnerable subjects, considering the well-known risk factors of diabetes and suggestions from clinical physicians (S2 Table), a case filter was listed. We filled each filter with participants who had T2DM, were ≥18 years old and were permanent residents in Tianjin. A final total of 229 files were prepared for downstream analysis. 30 individuals were excluded because their interview materials were incomplete.

### Participants enrolment

The most important one of participants enrolment principles was that the participant had worst situation (family environment, treatment conditions, funds, etc.) than others. All participants were recruited by field workers. Most of the participants were hospitalized patients. Other parts of the participants were interviewed by field workers at home.

### Data collection

A set of guidelines contained open questions and topics for conversation that would be raised during the semi-structured interview. The questionnaire guidelines were uploaded in supplemental materials.

To measure the participants’ socioeconomic status and their sociodemographic data, these data were gathered before or subsequent to the interview.

### Data analysis

#### Analysis process

All audio records were transcribed into text and imported into NVivo Software (Version 10) and analysed according to TCA [10]. Then, an initial code manual was developed via a within-group discussion. Cross-validation was performed after 2 or 3 transcripts were coded by members in each group. After the work check, the salient contents were extracted by coders and were shared at pre-set intervals. Themes were discussed within groups until consensus was reached regarding validity of the themes after the coding process. Then, specific preliminary social and cultural factors were drawn up based on themes.

The identified factors in each transcript and the corresponding sociodemographic information extracted from the self-filled questionnaires were combined to generate the vulnerability matrix, which was used for downstream analysis. The final code manual is shown in S3 Table.

#### Comment on ethics and IRB

This study has obtained approval from the Ethical Review Committee of Tianjin Medical University. The study complies with data protection legislation. All the participants signed an informed consent form before the interview, and their participation is voluntary and may be discontinued at any point. An identification number was assigned to each individual for the purposes of pseudonymisation. The participants’ names were not mentioned during the interview. All necessary materials, including declaration of consent and participants’ identity information, were accessible only to the members of the research teams. At the end of the interview, each participant received a gift from field workers as a form of thanks.

## Results

### Overview of all participants

All demographic information shown in Table 1. From Table 1, we could see that the participants’ average age was 56.36 years old. The majority of participants were female (53.7%). 41.8% of the females had given birth to only one child. 96.5% of the participants were ethnic Han, and 94.3% of them were married. The median Fasting blood glucose (FBG) of these participants was 8.1 mmol/L, the median 2 h PBG was 11.2 mmol/L, and the median glycosylated haemoglobin was 8.4%. The bodily form-related indicators were available for 60.7% of all participants, including weight (median: 70.0 kg), height (average: 166.3 cm), waistline (median: 90.0 cm), and BMI (median: 25.9). 74.1% of the participants suffered from complications, and 32.8% of the participants suffered from co-morbidities. The median duration of diabetes of the participants was 13.0 years. 70.3% participants suffered from high BMI. 60.7% participants were suffered from abdominal obesity.

**Table 1:** The demographic information of all participants.

Demographic information	All participants
N	229
Age, x±s	56.36±13.36
Sex, n (%)	
Male	106(46.3)
Female	123(53.7)
Illiteracy or educated primarily, n (%)	
No	7(3.1)
Yes	7(3.1)
Missing	215(93.9)
Ever pregnant, n (%)	
No	24(19.7)
Yes	98(80.3)
Missing (Except males)	107(46.7)
Number of birth given, n (%)	
1	41(41.8)
2	22(22.4)
3	11(11.2)
4	1(1.0)
5	1(1.0)
Missing (Except males)	47(48.0)
Ethnic, n (%)	
Han	221(96.5)
Hui	5(2.2)
Others	3(1.3)
Married, n (%)	
Yes	214(94.3)
No	6(2.6)
Devoiced	7(3.1)
Missing	2(0.9)
FBG, M(IQR) *	8.1(7.0–10.0)
2h PBG, M(IQR) **	11.2(9.3–14.0)
Glycosylated hemoglobin (%), M(IQR)	8.4(7.1–10.0)
Weight, M(IQR)	70.0(62.0–80.0)
Height, x±s	166.3±8.3
Waistline, M(IQR)	90.0(83.3–98.0)
Abdominal obesity, n (%) ***	
No	74(32.3)
Yes	139(60.7)
Missing	16(7.0)
BMI, M(IQR)	25.9(23.4–28.4)
High BMI, n (%) ****	
Yes	161(70.3)
No	68(29.7)
Complications	
No	59(25.9)
Yes	169(74.1)
Missing	1(0.4)
Type of complications	
Brain	25(14.79)
Kidney	49(28.82)
Eye	64(37.6)
Peripheral nerve	111(65.3)
Heart	70(41.2)
Foot	7(4.1)
Co-morbidities	
No	144(62.9)
Yes	75(32.8)
Missing	10(4.4)
Type of Co-morbidities	
Chronic respiratory disease	8(9.4)
Disable	4(4.7)
Heart and cerebral vessels	63(74.1)
Tumor	7(8.2)
Neuro-degeneration	2(2.4)
Duration of diabetes M(IQR)	13.0(1.0–29.0)

* Normal fasting blood glucose (FBG):3.9–6.1 mmol/L

** Normal 2 hours postprandial blood glucose (2h PBG): ≤7.8 mmol/L

*** Abdominal obesity: waistline males:≧90cm, female:≧85cm

****High BMI: ≥24

### Themes

Twelve themes that influenced patients’ vulnerability were identified (S4 Table). 29 social and cultural factors (S4 Table) and vivid examples were extracted from the transcripts, in line with thematic analysis. The themes were summarized based on topics mentioned most often by and concluded that specific situations influenced their wellbeing, health or emotions. The descriptions of each theme were shown as followings:

#### Theme 1: Financial constraints

Diabetic patients needed long-term care, which entailed great financial burden, so the patients who had low incomes or low retirement allowance, who were unemployed, or who entailed significant household expenses were financially vulnerable. We also found that medical insurance or high reimbursement from insurance can release participants from such a burden, but the process for application was quite complex.

*CCDTJ03057*: *“My salary is 2000 yuan a month*, *and my employer gives me another 80 yuan for insurance (that is 4% of my salary)*. *Therefore*, *only 960 yuan a year is available as subsidy (for medical services)*. *It is not enough*.*”*

*CCDTJ17213*: *“My husband has suffered from COPD*, *so he has to take medicine regularly*, *which costs us 1000 yuan twice a month*, *and only half of the costs can be reimbursed by insurance*.*”*

#### Theme 2: Severity of disease

Diabetes can result in various complications that are difficult to control, and diabetic symptoms also impact patients’ daily life, in particularly when it the condition is out of control.

Poor disease control could cause participants’ vulnerability. Some patients said that their blood sugar was not stable and some even had side effects after treatments.

*CCDTJ03112*: *“My most painful symptom is low blood sugar*. *When the medicine did not suit me*, *I would experience heart palpitations*.*”*

*CCDTJ17211*: *“After taking insulin shots*, *my skin broke into rashes*. *Then*, *my doctor changed my insulin*, *but the rashes were still there*. *My doctor and I do not know why they appeared*.*”*

#### Theme 3: Health literacy

Low health literacy limited participants’ ability to effectively manage their disease. A 76-year-old male patient (ID: CCDTJ04064) told us that he considered diabetes to be an incurable illness, and he also mentioned that he had heard that diabetes could be cured in the United States by an operation. Another 49-year-old female patient (ID: CCDTJ05032) thought that diabetes could be cured by removing islets (nesidiectomy). Some patients (CCDTJ13037, CCDTJ13090) told us diabetes is a killer—if a person has diabetes, he will be dead eventually. Some patients (CCDTJ18072, CCDTJ22078, and CCDTJ23038) even considered diabetes as a kind of cancer, which could not kill them but could torment them.

#### Theme 4: Health beliefs

How participants perceive health and health-related issues could impact their disease control. In our study, some patients were indifferent to diabetes and did not follow the doctor’s advice. A 66-year-old female participant (ID: CCDTJ01041) who suffered from severe diabetes complications said that she did not want to inject the insulin even though her doctor told her to do so, and she never shared her thoughts with her doctor.

Those who did not actively look for health knowledge (ID: CCDTJ05008, CCDTJ21001, etc.) was more likely to suffer severe disease than those who did so.

*CCDTJ21001*: *“When I found my blood sugar became higher*, *I did not go to hospital for treatment*. *I did not pay attention to it*. *My blood sugar value is not healthy*, *about 18*.*”*

Distrust of primary health services also becomes an obstacle in seeking medical services. Some patients (ID: CCDTJ18216, CCDTJ21121) distrusted the community hospital, even though it could provide professional services such as blood sugar monitoring and physical examinations. On the other hand, insisting on receiving healthcare in high-level hospitals generally costs time and money.

*CCDTJ18216*: *“I go to a specialized hospital to treat my diabetes instead of other hospitals*. *Doctors in community hospitals are not capable*, *their capacity is even worse than mine*. *So*, *when I have a disease*, *I always choose to go to a specialized hospital*.*”*

*CCDTJ21121*: *“I don’t go to a community hospital for treatment*. *I don’t trust them*. *And I am afraid that after their treatment I would be worse than before*.*” “I would rather spend more money to go to a general hospital than a community hospital*.*”*

#### Theme 5: Medical environment

Medical resources were unequally distributed in Tianjin, which made many patients feel that it was inconvenient to seek medical services. Patients (ID: CCDTJ01019, CCDTJ01107) complained about the lack of professional medical institutions and reliable hospitals near their communities.

*CCDTJ01019*: *“My community is very inconvenient in many aspects (including seeking medical advice)*. *A long time ago*, *I heard that a general hospital would open a branch hospital near my community*, *but up till now*, *this has not been true*.*”*

#### Theme 6: Life restrictions

After being diagnosed with diabetes, patients’ daily life was restricted, and some even experienced restriction at their work place. A 60-year-old female patient (ID: CCDTJ01107) said that after the diagnosis, she feels weak all the time, so she had to retire 2 years earlier than expected. Another patient (ID: CCDTJ18043) said that due to his diabetes, he could not enjoy his work as before. Injected insulin should be kept in the freezer, and therefore patients (ID: CCDTJ01107, CCDTJ03141) have to give up long-distance travel after being diagnosed.

*CCDTJ01107*: *“If the temperature is high*, *then I won’t go to travel*, *because I am afraid that the insulin would lose its efficacy because of the warm weather*.*”*

*CCDTJ01041*: *“Diabetes has changed my interests*. *I liked dancing before*, *but now*, *I cannot keep dancing*.*”*

#### Theme 7: Lifestyle change

Lifestyle is a well-known factor that influences disease control. However, changes in unhealthy lifestyle were difficult for most of the patients because they have to adhere to their customs and traditional choices. Other external factors also influence the participants’ choices. For example, health conditions limited participants’ capability to exercise routinely (CCDTJ07044, CCDTJ07165, etc.).

*CCDTJ07050*: *“We have a reunion every spring festival and mid-autumn day*, *my sisters and brothers come over*, *and we eat meat most of these times*. *Chicken*, *shrimp and crab meats are necessary*.*”*

*CCDTJ08062*: *“I like to eat fried foods*, *and I eat them every morning*.*”*

Some patients have irregular sleep patterns or poor sleep condition, from the medical aspect, which definitely impact patients’ recovery and emotions.

*CCDTJ01087*: *“Sleep quality is my biggest problem*, *I go to sleep at 11 pm and get up at 6 am every day*, *but during the sleep time*, *I barely have a deep sleep*, *sometimes I am even awake all night*.*”*

#### Theme 8: Time poverty

Living a busy life kept participant from seeking health services. Time poverty mainly resulted from overwhelming work and/or taking care of families. The time poverty we found had not caused stress for the participants.

*CCDT13194*: *“I have been told that there is not enough medicine for me today*, *but since I only have one hour of break time*, *I have no time to go to another hospital*, *so I probably will have no medicine at all in the next two weeks*.*”*

*CCDTJ01133*: *“I had hoped we could play Taiji together*, *either organized by ourselves or by neighbourhood committee*. *But this is unrealistic*, *because we all have work; those people who are my age or 1 or 2 years older than me*, *we have to work hard*.*”*

#### Theme 9: Mental condition

Many participants felt depressed, anxious, or nervous because of suffering from diabetes and related complications, especially at the time of diagnosis, although some of them said they have the confidence to control the disease. Some patients have suffered from diabetes for a long time, and they felt tired of taking pills and getting hospital treatments. These patients seemed negative and felt no hope for the future.

*CCDTJ18217*: *“I feel better now*, *I am always an optimist or I would not be myself today*, *I have overcome so many problems*. *But I know that I had some negative thoughts at the beginning of my diagnosis*. *I was afraid at that time*.*”*

*CCDTJ12187*: *“I do have any confidence in the future*, *I am 20*, *I have this disease*, *and I don’t have a decent job*, *why should I have confidence*? *I am not even sure whether I will live tomorrow*.*”*

#### Theme 10: Levels of support

Three aspects of support were related to diabetic patients’ vulnerability: community support, support from friends and family and social support. Participants who lived in a community with a poor environment (no sports or medical facilities, no open field for sports, etc.) were less likely to get exercise. Community-organized health knowledge propaganda and physical examinations were useful approaches for diabetes management; however, most of the participants said they have never received such support (CCDTJ01134, CCDTJ01135, etc.).

Family and friends play an important role in participants’ disease control. In families where the members barely cared about participants’ health and no one was willing to hear the participant’s narration of their disease, the participants might feel lonely and hopeless, and their disease condition would continually deteriorate.

We also found that some family members provided unconfirmed information to the participants, and this information was easily accepted since participants were more likely to believe their family members and friends than others.

Social support and community support were essential to patients’ resilience. Patients felt they were neglected by society because they were never provided any help in diabetes management.

*CCDTJ01134*: *“No*, *there is not any support for diabetes patients around our community*.*”*

*CCDTJ01135*: *“I have not heard that there have ever been organizations that provide health knowledge or any medical services such as blood sugar tests for us*.*”*

*CCDTJ18072*: *“I live alone and feel lonely*.*”*

*Doc*: *“Don’t your children care about you*?*” CCDTJ18072*: *“No*, *they don’t*.*”*

*CCDTJ01133*: *“I never received any help from society*, *and I never hear about such help*, *the government doesn’t care about us*. *Only my ward mate ever helped me*.*”*

#### Theme 11: Social integration

Social integration helps patients share information and knowledge and influences patients’ emotions as well. We found that a lack of social intercourse leads to negative emotions and a low-spirited life.

Although Chinese people attach importance to social integration, many participants would also like to maintain their privacy; especially when they experience difficult times, they always choose to suffer it alone. This behaviour hinders information sharing, and because their friends and family members never know about their health situations and real emotions, useful information cannot be delivered.

*CCDTJ13037*: *“I don’t go out*, *I don’t talk with others*, *and I don’t know their daily life*.*”*

*“………*..*no one introduces me to diabetes knowledge*.*”*

*CCDTJ03071*: *“I did not tell my family about my disease*, *I think I can handle it by myself*. *I do not think I need any help*, *I can deal with it*.*”*

#### Theme 12: Experience of transitions

Diet and dwelling environment were related to patients’ diabetes development. Participants who experienced transition from a life of poverty in their youth to a well-off life at middle age were more likely to live an irregular lifestyle, or to change their lifestyle. In addition, farmers who lived in the countryside and moved to cities, especially to the metropolis, also changed their lifestyle towards an “urbanized lifestyle”.

*CCDTJ22056*: *“Now our living standard has improved a lot*, *my diet has changed a lot*. *When I was young*, *all foods we ate had a fixed quantity*. *We did not have that much to eat*, *especially for meat*, *eating oil*, *fish or even tofu and vegetables*.*”*

*CCDTJ18218*: *“I used to live in the countryside and grew vegetables myself*, *but I never do that since I moved to Tianjin*.*”*

### Factors affecting all participants

We have identified 12 combined factors associated with participants’ vulnerability (Table 2), and 6 of them were found in more than half of the participants. These were severity of diseases (97.8%), low level of support (92.6%), lifestyle change (84.7%), experience of transitions (80.3%), life restriction (76.9%), and lack of medical resources (66.4%).

**Table 2:** Factors affecting all participants.

Factor	Factors descriptions	Participants
Low Income, Unemployment, No Medical Insurance/Low ratio reimbursement	Financial Constraints	79(34.5%)
Appear symptoms, complications, co-morbidities, high BMI, Poor disease control	Severity of Diseases	224(97.8%)
No/Low Wrong knowledge health literacy	Low Literacy	78(34.1%)
Perceived diabetes indifferently, Acquire Health Knowledge Passively, Distrust of primary health services	Adverse Health Belief	92(40.2%)
Needs not meet by Medical Services	Needs Not Met	152(66.4%)
Daily Life, Occupational Restriction	Life Restriction	176(76.9%)
Adhering to traditional or unhealthy diet, Lack of exercise, Low-quality sleep	Lifestyle Change	194(84.7%)
Healthcare seeking behavior were limited by work, Healthcare seeking behavior were limited by taking care of family issues	Time Poverty	75(32.8%)
Negative emotions towards diabetes, Negative emotions towards life	Negative mental Condition	56(24.5%)
Lack of community support, Lack of Friends and Family Support, Lack of Social Support	Low levels of Support	212(92.6%)
Low Degree of Integration, Faith in Suffering Alone	Low Degree of Integration	61(26.6%)
Diet, Dwelling Environment	Experience transformation	184(80.3%)

## Discussion

CCD Tianjin revealed the local vulnerabilities of patients with diabetes. We obtained 12 themes and 29 corresponding factors. The items involved physical, mental and social losses that made life more limited and uncertain.

Previous studies have shown that obesity has adverse influence on diabetes patients. H.E. Bays et al. ^[^^8^^]^ found that an increase in BMI was associated with an increase in prevalence of diabetes. An advance study ^[^^9^^]^ has shown that diabetic complications had an impact on patients’ quality of life. Our findings were consistent with this study in that the patients with complications were more vulnerable than those who were complication-free.

In terms of blood glucose monitoring, knowledge improvement is a significant promoter [10,11]. In our study, many participants, especially the illiterate and less-educated individuals, did not know the correct definition of diabetes, let alone how to fully manage their disease. This result confirmed that there are still knowledge deficiencies in Chinese adults with diabetes[11]. Therefore, it is worth attempting to enhance diabetics’ knowledge about diabetes and to strengthen the diabetes propaganda and education, in particular for the individuals who are less educated in Tianjin.

We found that having diabetes may bring additional financial burdens to patients and their families, who have to balance the cost of obtaining healthcare against competing financial demands, such as paying for food[12]. Diabetes and its resulting disabilities can lead to difficulties in working or seeking employment. In addition, the long-term care and high cost of healthcare have major impacts on household income, potentially leading to vicious cycles of poverty and illness [13]. Having health insurance may alleviate some of the financial pressures associated with diabetes and the obstacles to obtaining medical care, thereby increasing patients’ access to care[12]. Meanwhile, reimbursement from the Outpatient Special Disease Insurance (ODSI) in Tianjin can further eliminate some of the financial barriers for patients with diabetes.

Almost all participants in our study had unhealthy lifestyles before diagnosis, and this may be the main reason causing the onset of diabetes. Many studies have shown that lifestyle interventions through physical activity and healthy diet are generally cost-effective for controlling diabetes [14]. However, to change one’s original lifestyle is not easy, even though most of the participants were aware of the necessity. Further studies in exploring effective approaches to promote healthy lifestyles for patients with diabetes in Tianjin are needed.

Of our participants, 92.6% reported lack of support either from family, community or society. Given that family members have been recognized as an important part of the support network for people with diabetes[15], the implementation of interventions regarding diabetes knowledge and psychological health for both diabetes patients and their families is highly suggested. We also found that the social integration among neighbours, friends and ward mates has significant impacts on the participants’ resilience. Therefore, preventive interventions that target high-risk individuals and their social network members may improve the effectiveness of disease control [16–18].

Our results indicated that the participants complained about lack of medical resources in their community. Patients avoid primary healthcare services, seeking unnecessary high-level medical services for preventable conditions[19]. Our government has realized this problem and has begun to focus on the development of primary healthcare services. Patients also need support from the workplace and society.

We found that experiencing environmental transitions may have significant effects on the development of diabetes[20]. The high susceptibility to urban diabetes might be caused by the newly adopted lifestyle and the low resistance to its diabetogenic effects [21]. It is also well-known that the prevalence of diabetes increases with rapid migration and urbanization [22,23], which result in the transition from rural to urban lifestyle [24–26]. We found that people who used to live in the countryside and moved to town abandon their traditional lifestyles to adopt a diet rich in saturated animal products, salt, sugars and fats, and they also decrease physical activity.

A number of participants who thought diabetes was untreatable felt depressed, even anxious. We also found that the patients who had negative attitudes towards diabetes treatment could not control their blood glucose well. Negative emotions and poor glycaemic control could interact with each other[27]. Further evidence is needed to illustrate this relationship in the Tianjin setting.

This study has several limitations. The participants were enrolled by field workers who worked in hospitals, which meant that these field workers would catch more information from those samples than from general patients with diabetes. Similarly, since most of our participants were retired, their experiences and views may not match those of younger patients.

## Conclusion

To the best of our knowledge, this is the first large-scale, qualitative investigation examining the combined social and cultural factors central to the development and experience of T2DM in Tianjin. People with diabetes have certain characteristics regarding vulnerability. Based on our findings, specific interventions targeting individual patients, family, community and society are needed to improve diabetes control, and patients’ mental health care and general improvement of living conditions are also needed.

## Supporting information

S1 Table Field worker enrolment list. The hospitals we trained interviewers.
(DOCX)

S2 Table Case filter and its definition. The principle of enrol participants.
(DOCX)

S3 Table Coding manual. The principle of coding.
(DOCX)

S4 Table Themes and factors of vulnerability of diabetic patients in Tianjin. The final themes and factors of vulnerability affected diabetic patients.
(DOCX)

S1 File Vulnerability assessment field summary (English version). (DOCX)

S2 File Interview outline (survey questions). (PDF)
