# Supplementary material for: Assessment of factors affecting diabetes management in the City Changing Diabetes (CCD) study in Tianjin
Source: PLoS One. 2019 Feb 12;14(2):e0209222. doi: 10.1371/journal.pone.0209222 (PMC6372168; doi:10.1371/journal.pone.0209222)
Supplement: S1 Table — The hospitals we trained interviewers. (DOCX) [file pone.0209222.s001.docx]

**S1 Table Field worker enrolment list**

| **Hospital Level** | **Hospital Name** |
| --- | --- |
| Grade-I Hospital | Tianjin Medical University General Hospital |
|  | Tianjin Chest Hospital |
|  | Tianjin 4th Center Hospital |
|  | Tianjin First Center Hospital |
|  | Tianjin Academy of Traditional Chinese Medicine Affiliated Hospital |
|  | Tianjin People’s Hospital |
|  | Tianjin Third Central Hospital |
|  | Tianjin Hospital |
|  | Second Affiliated Hospital of Tianjin University of TCM |
|  | Second Affiliated Hospital of Tianjin Medical University |
|  | Tianjin Medical University Metabolic Disease Hospital |
|  | Tianjin Fifth Central Hospital |
|  | TEDA Hospital |
| Grade-II Hospital | Tianjin Second Hospital |
|  | Tianjin Armed Police Corps Hospital |
