# Supplementary material for: Assessment of factors affecting diabetes management in the City Changing Diabetes (CCD) study in Tianjin
Source: PLoS One. 2019 Feb 12;14(2):e0209222. doi: 10.1371/journal.pone.0209222 (PMC6372168; doi:10.1371/journal.pone.0209222)
Supplement: S2 Table — The principle of enrol participants. (DOCX) [file pone.0209222.s002.docx]

**S2 Table Case filter and its definition**

| **Case filter** | **Definition** |
| --- | --- |
| High BMI | BMI≥24 |
| Duration of diabetes | At least 10 years |
| Diabetes complications/co-morbidities | Have at least one kind of diabetes complications or co-morbidities |
| Health insurance | No basic insurance, no urban basic insurance or no any commercial health insurance |
| Employment status | Unemployment |
| Below poverty level | Per capita income less than￥705 for urban residence, and less than￥540 for rural residents. |
| Body size and physical characteristics | Waistline: Male≥90cm, Female≥85cm |
| Education background | Primary or illiteracy |
| Physical activity level | Low (no exercise etc.) |
