# Supplementary material for: Assessment of factors affecting diabetes management in the City Changing Diabetes (CCD) study in Tianjin
Source: PLoS One. 2019 Feb 12;14(2):e0209222. doi: 10.1371/journal.pone.0209222 (PMC6372168; doi:10.1371/journal.pone.0209222)
Supplement: S3 Table — The principle of coding. (DOCX) [file pone.0209222.s003.docx]

**S3 Table Coding manual**

| **Code** | **Definition** | **Description** |  |
| --- | --- | --- | --- |
| B1 | Severity of Disease | Information about how the severity of symptoms impacts the person in daily life and in medical decision making | B1.a. Care seeking and care decision making. The severity of diabetes impacts the level of care facility the person is using. |
|  |  |  | B1.b. Co-morbidities(excluding diabetes) and complications are present and have an impact. |
|  |  |  | B1.c. The person is impacted in some way by their diabetes and associated symptoms in their daily life |
|  |  |  | B1.d. The person does not experience diabetes symptoms (or is not impacted in daily life) |
| C1 | Food Traditions and Habits | Information about how local food traditions and personal diet habits impact a person’s health and diabetes management | C1.a. Local food traditions impact the person’s food choices |
|  |  |  | C1.b. Personal diet habits impact the person’s food choices |
| C2 | Health Beliefs | Information about how health beliefs impact a person’s ability to make good decisions and manage their diabetes well | C2.a. The person’s understanding of their health and illness and/ or diabetes is not aligned with biomedical understanding |
|  |  |  | C.2.b. The person’s diabetes care and management are impacted by their health beliefs |
|  |  |  | C2.c. Description of causes of diabetes (including ‘folk beliefs’) |
| C3 | Religion and Spirituality | Information about how R&S impacts a person’s behavior and attitude/vulnerability to diabetes |  |
| E1 | Environmental Givens | Information about how local environmental givens impact diabetes vulnerability | E1.a. Pollution and health; exercise and being outside |
|  |  |  | E1.b. Environment: access to parks, outside space, etc. |
|  |  |  | E1.c. Medical environment |
| E2 | Food Safety | Information about how perceptions about available foods impact a person’s eating habits and diabetes vulnerability |  |
| P1 | Policy Impact | Information about the influence of local medical insurance policy on diabetes care and behavior | P1.a. Medical insurance policy impact on the diagnosis and treatment of diabetes |
| S1 | Financial Situation | Information relating to financial situation that describes how finances impact diabetes vulnerability | S1.a. Income: the person’s income impacts what she or he is able to do that is relevant to their health |
|  |  |  | S1.b. Insurance: the local insurance situation impacts this person’s financial situation. Any information about the local percentage of pay and treatment choice |
|  |  |  | S1.c. Daily and other diseases’ expenditures: the person’s daily life and other diseases’ expenditures impact their financial situation |
|  |  |  | S1.d. Diabetes and its complications’ expenditures |
|  |  |  | S1.e. There are dependents or there are other significant expenses |
|  |  |  | S1.f. Impact of FS: the person’s sense of wellbeing is impacted by their financial situation (maybe they are anxious, depressed, stressed) |
| S2 | Employment Status | Information relating to financial situation that describes how employment status impact diabetes vulnerability | S2.a. Retired |
|  |  |  | S2.b. Self-employed(including farmers) |
|  |  |  | S2.c. Working |
|  |  |  | S2.d. Unemployed(including student) |
|  |  |  | S2.e. Impact of ES on mental condition |
|  |  |  | S2.f. Impact of ES on financial status |
| S3 | Level of Education | Different levels of education |  |
| S4 | Lifestyle | Information about lifestyle choices that impact diabetes vulnerability | S4.a. Levels of exercise impact physical health and mental condition |
|  |  |  | S4.b. Sleep status impact physical health and mental condition |
| S5 | Beliefs and Literacy towards Diabetes | Information about the person’s health beliefs and level of health literacy towards diabetes | S5.a. The health literacy towards diabetes |
|  |  |  | S5.b. The person’s understanding of the high-risk groups of diabetes (the specific examples to describe) |
| S6 | Level of Support | Information about the support level of the patient from various aspects | S.6.a. Community support |
|  |  |  | S.6.b. Familial support |
|  |  |  | S.7.c. Social support |
| SC1 | Past Experience | Information about the impact of past experiences on diabetes care and management |  |
| SC2 | Medication and Care | Information about the person's medication and care for general health concerns and diabetes | SC2.a.Normal medication organization |
|  |  |  | SC2.b. Self-medication(excluding normal hospitals and doctors) |
| SC3 | Time Poverty | Information about the person's availability of time | SC3.a. The majority of available time is spent with work and work-related matters(including impact of employment status) |
|  |  |  | SC3.b. The person is the main carer for relatives (e.g. elderly parents) |
|  |  |  | SC3.c. The person reports not having enough time at their disposal for health-related activities |
| SC4 | Care Provision | Information about care provision and treatment impact a person’s health and diabetes vulnerability | SC4.a. Care provision and treatment is guided by trust in specific people |
| SC5 | Social Integration | Information about levels of social integration and associated health benefits and/or negative impact on health and diabetes care | SC5.a. Levels of social integration and the impact on sharing of information/advice/medication |
|  |  |  | SC5.b. Suffer alone |
| SC6 | Experience of Diabetes | Information about how a person experiences having diabetes | SC6.a. The length between onset of symptoms to diagnosis so far |
|  |  |  | SC6.b. How do post-diagnosis impact person’s attitude and health behavior |
| SC7 | Character and Mental Condition | Information about the person's character and mental Condition | SC7.a. Self-description of the person’s mental condition |
|  |  |  | SC7.b.Others’ status impacts the person’s mental condition |
|  |  |  | SC7.c. The person’s character impacts diagnosis and treatment of diabetes |
| SC8 | Attitude towards Diabetes | Impact of attitude towards diabetes prevention and treatment(positive/negative) | SC8.a. Positive attitude of diabetes |
