# Supplementary material for: Assessment of factors affecting diabetes management in the City Changing Diabetes (CCD) study in Tianjin
Source: PLoS One. 2019 Feb 12;14(2):e0209222. doi: 10.1371/journal.pone.0209222 (PMC6372168; doi:10.1371/journal.pone.0209222)
Supplement: S4 Table — The final themes and factors of vulnerability affected diabetic patients. (DOCX) [file pone.0209222.s004.docx]

**S4 Table Themes and factors of vulnerability of diabetic patients in Tianjin**

| **Theme** | **Sub-theme 1** | **Sub-theme 2** | **Corresponding factors** |
| --- | --- | --- | --- |
| Biological risk factors |  |  | Age, Sex, Racial, Marriage Status, Household Register, Family History of Diabetes |
| Financial  Constrain | Incomes or Retirement Allowance |  | Low Income |
|  | Employment |  | Unemployment |
|  | Medical Insurance | Ratio of Reimbursement | No Medical Insurance/Low reimbursement ratio |
|  |  | Possession of medical insurance |  |
|  | Family expenditure |  | Significant family expenditure |
| Severity of Diseases | Complications/Co-morbidities |  | Appear Symptoms, Complications, Combidities |
|  | Appearance of Diabetic Symptoms |  |  |
|  | Disease Control |  | Poor disease control |
| Health Literacy | Health Literacy |  | Low Literacy |
| Health Beliefs | Perceive diabetes treatment |  | Perceived diabetes indifferently |
|  | Attitude to acquire Health Knowledge |  | Acquire Health Knowledge Passively |
|  | Perceived primary health services |  | Distrust of primary health services |
| Medical environment | Needs meet by Medical Services |  | Needs not meet by Medical Services |
| Life restriction | Daily Life Behaviors |  | Limited Daily Life Behaviors |
|  | Occupational Restriction |  | Occupational Restriction |
| Lifestyle Change | Food choice |  | Adherence to the traditional or unhealthy food choice |
|  | Exercise |  | Lack of Exercise |
|  | Quality of sleep |  | low-quality sleep |
| Time Poverty | Limitation of Healthcare seeking behavior |  | Healthcare seeking behavior were limited by work/taking care of family issues |
| Mental Condition | Emotions towards diabetes treatment or life |  | Appearance of negative emotions towards diabetes treatment or life |
| Levels of Support | Community Support | Community Environment | Lack of community support |
|  |  | Support for Diabetes |  |
|  | Friends and Family Support | Provided Cares | Lack of Friends and Family Support |
|  |  | Provide the inaccurate information |  |
|  | Social Support |  | Lack of Social Support |
| Social Integration | Degree of Social Integration |  | Low Degree of Social Integration |
|  | Interpersonal Belief |  | Faith in Suffering Alone |
| Experience transitions | Diet |  | Diet transformation |
|  | Dwelling Environment/Place of Residence |  | Dwelling Environment/Place of Residence transformation |
