# Supplementary material for: Assessment of factors affecting diabetes management in the City Changing Diabetes (CCD) study in Tianjin
Source: PLoS One. 2019 Feb 12;14(2):e0209222. doi: 10.1371/journal.pone.0209222 (PMC6372168; doi:10.1371/journal.pone.0209222)
Supplement: S1 File — (DOCX) [file pone.0209222.s005.docx]

**Vulnerability Assessment Field Summary--Tianjin**

| **Date** |  |
| --- | --- |
| **Interview ID** |  |
| **Researcher ID and signature** |  |

**The filter for participant recruitment**

|  |
| --- |

**Please flag any important issues in red here:**

|  |
| --- |

Make sure to cover at least the following questions, and feel free to expand and be creative with your summary.

1. **In a few sentences, describe the setting in which the interview took place.**

- Where did the interview take place?
- How did you get here?
- Did you notice anything unusual or special on the way to the interview location?
- If you went to somebody’s house, describe it in a few sentences.
- Overall impression of the neighbourhood:
  - Were there mostly houses/shops/restaurants etc.?
  - Did it seem affluent/poor/mixed/‘up-and-coming’, etc.?
  - Were any people outside, sitting and watching the street? Walking about? Jogging, cycling? Children playing outside? Anything else?

|  |
| --- |

1. **In a few sentences, describe your interview participant.**

- What was her or his overall appearance:
  - How were they dressed?
  - Did you have the impression they made a special effort for the interview? Or the contrary?
- Did the person seem healthy?
- Did the person strike you generally as content/happy/unhappy/sad/anxious, etc?

|  |
| --- |

1. **Please describe the interview itself.**

- Was there anything particularly noteworthy about the interview?
  - Either something the person said, or how they said it.
  - Something that you noticed that was not openly talked about.
- Did the person show you photos/ medication/ anything else? Please describe/ provide example.
- Based on your impression:
  - What would you say was the main problem the person was facing regarding their health/ diabetes?
  - What was good in their life regarding their health/ diabetes?
  - What might make this person particularly vulnerable to suffering from the consequences of their condition?
  - Is there something that might help them right away?

|  |
| --- |

1. **Did the participant seem to be interested in the CCD project and motivated to provide information?**

**the Chinese version do not have this section**

- Provide example (describe body language, or quote from interview).
- Did the participant ask any questions about the project?
- Provide an example, and response given.

|  |
| --- |

1. **If the person said something that struck you as particularly interesting or important please note it down here.**

- You can refer to the time it occurs in the interview or quote.

|  |
| --- |

1. **Did the person suggest there were others more vulnerable to suffering from ill-health and its complications?**

- If yes, please provide details here**.**

|  |
| --- |

1. **Please add anything else that you think might be important for us to know.**

|  |
| --- |
