# Supplementary material for: Assessment of factors affecting diabetes management in the City Changing Diabetes (CCD) study in Tianjin
Source: PLoS One. 2019 Feb 12;14(2):e0209222. doi: 10.1371/journal.pone.0209222 (PMC6372168; doi:10.1371/journal.pone.0209222)
Supplement: S2 File — (PDF) [file pone.0209222.s006.pdf]

糖尿病脆弱性评估 — 目前的情况 (F)

(F1) 帮助本质

(F1.1) 被确诊为糖尿病后，您是否接受过任何形式的帮助?

是 否

(F1.1) 如果您的答案为“是”，您都接受过哪些帮助?

(F1.1) 如果您的答案为“是”，谁提供的这些帮助?

(F1.1) 如果您的答案为“是”，您第一次因糖尿病接受帮助发生在什么时候?

(F1.1) 如果您的答案为“是”，您接受帮助的时间有多久?

(F1.1) 如果您的答案为“否”，您未获得任何帮助的原因是什么?

(F1.2) 您是否使用医疗保险?

是 否

(F1.2) 如果您的答案为“是”，您参加的是什么医疗保险? 糖尿病的报销比例是多少?

(F1.2) 如果您的答案为“否”，您为什么没有医疗保险?

(F1.2) 如果您的答案为“否”，您是否可参加所在地的医疗保险?

(F1.2) 如果您的答案为“否”，您是否为所获得的糖尿病帮助付费?

(F1.3) 您是否知道糖尿病患者可得到其他形式的帮助?

是 否

(F1.3) 如果您的答案为“是”，是什么原因使您不能得到这些帮助?

(F1.4) 您的家庭/家人是否因您的糖尿病接受过任何形式的帮助? ___

是 否

(F1.4) 如果您的答案为“是”，他们都接受过哪些帮助?

(F1.4) 如果您的答案为“否”，请详细说明

(F1.4) 如果您的答案为“否”，您认为需要哪些帮助?

(F1.5) 如果您的家庭/家人糖尿病他们是否接受过任何形式的协助?

是 否

(F1.5) 如果您的答案为“是”，他们都接受过哪些帮助?

(F1.5) 如果您的答案为“否”，是否因为您是家中唯一患有糖尿病的人?

糖尿病脆弱性评估 — 目前的情况（F）

（F2）疗效/影响的持续性

（F2.1）您接受的帮助对控制糖尿病是否长期有效？

是　　否

（F2.1）如果您的答案为“是”，请详细说明

（F2.1）如果您的答案为“否”，请详细说明

（F2.2）您接受的糖尿病帮助对您整体的健康感知是否有持久的影响？

是　　否

（F2.2）如果您的答案为“是”，请详细说明

（F2.2）如果您的答案为“否”，请详细说明

糖尿病脆弱性评估 — 目前的情况（F）

（F3）满足需求

（F3.1）您接受的协助是否可以满足您的要求？

是　　否

（F3.1）如果您的答案为“是”，请详细说明

（F3.1）如果您的答案为“否”，请详细说明

（F3.2）您认为您的糖尿病需求是否可以在未来得到满足？

是　　否

（F3.2）如果您的答案为“是”，请详细说明

（F3.2）如果您的答案为“否”，请详细说明

(F4) 获取公平性

(F4.1) 您是否知道任何其他人（非您的家庭成员）接受过糖尿病协助?

是　否

(F4.1) 如果您的答案为“是”，他们接受的协助与您接受的协助相比怎么样?

(F4.1) 他们是否受益于接受的协助?

(F4.2) 一般情况下，您认为您所在的地方是否可以为糖尿病患者提供良好的协助?

是　否

(F4.2) 如果您的答案为“是”，请详细说明

(F4.2) 如果您的答案为“否”，请详细说明

(F5) 健康传播

(F5.1) 是否有人向您介绍过糖尿病的相关知识?

是　否

(F5.1) 如果您的答案为“是”，与糖尿病有关的最有用的信息是谁给您的?

(F5.1) 如果您的答案为“否”，您自己是否试图寻找过糖尿病资料?

(F5.1) 如果您的答案为“否”，您自己是否能够找到令您满意的糖尿病资料?

(F5.2) 您接受的最有用的糖尿病信息是什么?

?

糖尿病脆弱性评估 — 目前的情况 (F)

(F6) 提供协助

(F6.1) 您生活的地方是否有任何（公益性的）社会团体向糖尿病患者提供帮助?

是 | 否

(F6.1) 如果您的答案为“否”，您如此认为的理由是什么？

(F6.1) 如果您的答案为“是”，是哪种机构？

(F6.1) 如果您的答案为“是”，您是否去该机构或接受他们的服务？

- (F6.1) 如果不是，为什么不呢？
- (F6.1) 如果您的答案为“是”，您接受过哪些服务？

(F6.2) 在您生活的地方，您是否知道任何其他人向糖尿病患者提供帮助?

是 | 否

(F6.2) 如果您的答案为“是”，他们是谁？

(F6.2) 如果您的答案为“是”，您是否曾接受过他们的服务？

(F6.2) 如果您的答案为“是”，请详细说明

(F6.2) 如果您的答案为“否”，您如此认为的理由是什么？

(F6.3) 您经常去看糖尿病的医疗机构是什么?

是 | 否

(F6.3) 如果您的答案为“是”，请详细说明

糖尿病脆弱性评估 — 目前的情况 (F)

(F7) 综述

(F7.1) 您想获得什么样的糖尿病协助?

?

糖尿病脆弱性评估 — 社区领域 (F)

(C1) 社区

(C1.1) 您是否有自己的朋友圈?

是 否

(C1.1) 如果您的答案为“是”，请您描述一下您的社区?

(C1.1) 如果您的答案为“否”，请详细说明

(C1.2) 您是否为当地土生土长?

是 否

(C1.2) 如果您的答案为“否”，请详细说明

(C1.3) 您生活的地方，糖尿病患者是否经常聚在一起锻炼?

是 否

(C1.3) 如果您的答案为“是”，您是否参加任何此类的活动? (C1.4) 如果您的答案为“是”，请详细说明

(C1.3) 如果您的答案为“否”，请详细说明

(C1.4) 您生活的地方，糖尿病患者是否聚在一起帮助周围的糖尿病患者?

是 否

(C1.4) 如果您的答案为“是”，您是否参加任何此类的活动? (C1.5) 如果您的答案为“是”，请详细说明

(C1.4) 如果您的答案为“否”，请详细说明

(C1.5) 您周围的人是否还有上述未提及的其他方式彼此帮助?

是 否

(C1.5) 如果您的答案为任何此类的活动?
(C1.5) 如果您的答案为“是”，请详细说

(C1.5) 如果您的答案为“否”，请详细说明

(C1.6) 您认为是否可以采取更多措施改善社区组织以帮助糖尿病患者?

是 否

(C1.6) 如果您的答案为“是”，您认为应该采取什么措施帮助糖尿病患者改善社区组织?

说明

糖尿病脆弱性评估 — 社区领域（F）

（C2）风俗和传统

（C2.1）您所在的社区是否有任何传统和习俗促进大家相互联系？

是　否

（C2.1）如果您的答案为“是”，请详细说明

（C2.1）如果您的答案为“是”，这些合作是短期还是长期的？

（C2.1）如果您的答案为“是”，这些合作是否仅限于您生活的地方？

（C2.2）您所在的社区，是否有任何风俗或传统影响人们的饮食习惯？

是　否

（C2.2）如果您的答案为“是”，请详细说明

（C2.3）您认为您所在社区的人们是否按照传统的生活方式生活？

是　否

（C2.3）如果您的答案为“是”，请详细说明

（C1.3）如果您的答案为“否”，请详细说明

（C2.4）您认为您所在社区的风俗和传统是否正在改变？

是　否

（C2.4）如果您的答案为“是”，请详细说明

（C2.5）是在不久前才发生的变化吗？

是　否

C2.6）如果您的答案为“是”，请详细说明

（C2.6）您所在的社区，通常人们向谁咨询健康问题

？

（C2.7）您到哪里咨询您的健康问题？

？

糖尿病脆弱性评估 — 社区领域 (F)

(C3) 营养

(C3.1) 您所在的社区，人们平日主要吃什么？

?

(C3.2) 您认为您和家人的平时主食是什么？

?

?

(C3.3) 您所在的社区，您认为人们庆祝时的主要吃什么？

?

(C3.4) 您认为您和家人庆祝时的主食是什么 ?

?

(C3.6) 您是否自己种植食物？

是 否

(C3.6) 如果您的答案为“是”，为什么？

(C3.6) 如果您的答案为“是”，您通常吃多少自己种植的粮食？

(C3.6) 如果您的答案为“否”，为什么？

(C3.7) 人们最常去哪儿买食品、蔬菜和杂货?

?

(C3.8) 您最常去什么地方买食品、蔬菜和杂货?

?

(C3.9) 人们最常去哪儿吃饭?

?

(C3. 10) 在您所在的社区，您最常去哪儿吃饭？

?

(C3. 11) 在您所在的社区，一周内人们在外就餐几次？

?

(C3. 12) 一周内您在外就餐几次？

?

(C3. 13) 您家谁负责准备食物？

?

糖尿病脆弱性评估 — 社区领域 (F)

(C4) 设施

(C4. 1) 在您所在的社区，人们是否有免费的饮用水？

是 | 否

(C4. 1) 如果您的答案为“是”，他们是在家获得饮用水的吗（从水龙头?）

(C4. 1) 如果您的答案为“否”，人们口渴的时候通常喝什么？

(C4. 1) 您工作的地方邻近住地吗？

是 | 否

(C4. 2) 如果您的答案为“是”，您怎么去您工作的地方？

(C4. 2) 如果您的答案为“否”，您去工作的地方有多远？
(C4. 2) 您怎么去上班？

(C4. 3) 您喜欢您生活的地方吗？

是 | 否

(C4. 3) 如果您的答案为“是”，请详细说明

(C4. 3) 如果您的答案为“否”，请详细说明

糖尿病脆弱性评估 — 社区领域 (F)

(C5) 锻炼

(C5. 1) 例如，您所在的社区里是否有公共区域供人们踢足球、打网球等或到公园里散步和跑步?

是 | 否

(C5. 1) 如果您的答案为“是”，请详细说明 (C5. 1) 如果您的答案为“是”，人们是否经常使用这些区域?
(C5. 1) 如果您的答案为“是”，请详细说明
(C5. 1) 如果您的答案为“是”，您自己经常使用它们吗?

(C5. 1) 如果您的答案为“否”，为什么不呢?

(C5. 2) 您所在社区的人们通常都进行哪些体育活动?

?

(C5. 3) 您都进行哪些体育活动?

?

(C5. 4) 您喜欢这些活动吗?

是 | 否

(C5. 4) 如果您的答案为“是”，请详细说明

(C5. 4) 如果您的答案为“否”，请详细说明

(C5. 5) 您认为自己是一个积极锻炼的人吗?

是 | 否

(C5. 5) 如果您的答案为“是”，请详细说明

(C5. 5) 如果您的答案为“否”，请详细说明

糖尿病脆弱性评估 — 脆性领域 (F)

(V1) 疾病认知/诠释

(V1.1) 请说明您对糖尿病的理解

?

(V1.2) 以您的观点来看，您认为哪些人最有可能患糖尿病?

?

(V1.3) 以您的观点来看，什么原因使人容易患上糖尿病?

?

(V1.4) 以您的观点来看，您认为什么样的糖尿病患者受到的伤害最大?

?

(V1.5) 以您的观点来看，您认为糖尿病最棘手的症状是什么?

?

(V1.6) 以您的观点来看，您认为糖尿病应该如何预防?

?

糖尿病脆弱性评估 — 脆性领域 (F)

(V2) 标准脆性指标

(V2.1) 您目前工作吗?

是　否

(V2.1) 如果您的答案为“是”，您的工作是长期的吗？(V2.1) 如果您的答案为“是”，您接受过单位福利吗？

(V2.1) 您接受过哪些单位福利？

(V2.1) 如果您的答案为“否”，请详细解释

(V2.1) 如果您的答案为“否”，您接受过政府福利吗？

(V2.1) 您接受过哪些政府福利？

(V2.2) 你是否独自生活?

是　否

(V2.2) 如果您的答案为“否”，请描述和您住在一起的人

(V2.3) 您是否需要照顾其他人?

是　否

(V2.3) 如果您的答案为“是”，此人是否也是糖尿病患者？

(V2.4) 与生活在同一社区的其他人相比，您是否觉得在经济上有保障？

是　否

(V2.4) 如果您的答案为“是”，请详细说明

(V2.4) 如果您的答案为“否”，请详细说明

(V2.5) 您认为您的基本生活需要是否得到解决?

是　　否

(V2.5) 如果您的答案为“是”，请详细说明

(V2.4) 如果您的答案为“否”，请详细说明

(V2.6) 您认为您是否能够控制糖尿病?

是　　否

(V2.6) 如果您的答案为“是”，请详细说明

(V2.6) 如果您的答案为“否”，请详细说明

(V2.6) 如果您的答案为“否”，什么能帮您更好地控制病情?

(V2.7) 您能负担得起糖尿病的治疗费吗?

是　　否

(V2.7) 如果您的答案为“否”，请详细说明

糖尿病(基因)脆弱性评估 — 脆弱性领域 (F)

(V3) 安全

(V3.1) 您所在的社区是否安全?

是　　否

(V3.1) 如果您的答案为“是”，请详细说明

(V3.1) 如果您的答案为“否”，请详细说明

(V3.2) 您居住的地方，一个人独自锻炼安全吗?

是　　否

(V3.2) 如果您的答案为“是”，您这样做安全吗? (V3.2) 请详细说明

(V3.2) 如果您的答案为“否”，请详细说明

糖尿病脆弱性评估 — 脆弱性领域 (F)

(V4)信任与动因

(V4.1) 您最信任什么人/途径为您提供一般性健康咨询?

?

(V4.2) 请告诉我您最信任/最不信任谁来进行糖尿病护理?

?

(V4.3) 患有糖尿病是否改变了您对生活的看法?

是 否

(V4.3)如果您的答案为"是"，请详细说明

(V4.3)如果您的答案为"否"，请详细说明

(V4.4) 患有糖尿病是否改变您的生活乐趣?

是 否

(V4.4)如果您的答案为"是"，请详细说明:

(V4.4)如果您的答案为"否"，请详细说明:

(V 4.5) 您是否对未来充满信心?

?

糖尿病脆弱性评估 — 脆弱性领域 (F)

(V5) 其他问题

(V5.1) 您是否知道还有其他无人照顾的糖尿病患者?

是 否

(V5.1) 如果有，他们是谁?

(V5.2) 就糖尿病经历，您还有其他情况要说明的吗?

?

谢谢

英国伦敦大学学院人类学
系 © A. 大卫 内皮尔 2014
年邮箱:
d.napier@ucl.ac.uk
